# Searching for the Best Treatment for Ramp Lesions: A Systematic Review and Network Meta-Analysis

**DOI:** 10.7759/cureus.41651

**Published:** 2023-07-10

**Authors:** Felipe Marin, Julio Soto, Maximiliano Barahona, Roberto Negrin

**Affiliations:** 1 Department of Orthopaedics, Clínica Las Condes, Santiago, CHL; 2 Department of Orthopaedics, Hospital Clínico Universidad de Chile, Santiago, CHL

**Keywords:** meniscal peservation, meniscal repair, medial meniscus, ramp lesion, knee

## Abstract

Ramp lesions are a common occurrence in patients with anterior cruciate ligament (ACL) tears. These lesions can be difficult to diagnose due to their concealed nature, and their treatment is crucial due to the stabilizing function of the medial meniscocapsular region. The optimal treatment option for ramp lesions varies depending on the size and stability of the lesion. The purpose of this study was to evaluate the best treatment option for ramp lesions based on the stability of the lesion, including no treatment, biological treatment, and arthroscopic repair. We hypothesize that stable lesions have a favorable prognosis with techniques that do not require the use of meniscal sutures. In contrast, unstable lesions require appropriate fixation, either through an anterior or posteromedial portal. This study is a systematic review and meta-analysis with a level of evidence IV. The study used Preferred Reporting Items for Systematic Reviews and Meta-Analyses (PRISMA) guidelines for a systematic review of clinical studies reporting outcomes of ramp lesion treatment. The PubMed/MEDLINE database was searched using Mesh and non-Mesh terms related to ramp lesions, medial meniscus ramp lesions, and meniscocapsular injuries. The inclusion criteria encompassed clinical studies in English or Spanish that reported the treatment of ramp meniscal lesions, with a follow-up of at least six months and inclusion of functional results, clinical stability tests, radiological evaluation, or arthroscopic second look. The analysis included 13 studies with 1614 patients. Five studies distinguished between stable and unstable ramp lesions using different criteria (displacement or size) for assessment. Of the stable lesions, 90 cases received no treatment, 64 cases were treated biologically (debridement, edge-curettage, or trephination), and 728 lesions were repaired. There were 221 repaired unstable lesions. All different methods of repair were registered. In stable lesions, three studies were included in a network meta-analysis. The best-estimated treatment for stable lesions was biological (SUCRA 0.9), followed by repair (SUCRA 0.6), and no treatment (SUCRA 0). In unstable lesions, seven studies using International Knee Documentation Committee Subjective Knee Form (IKDC) and 10 studies using Lysholm for functional outcomes showed significant improvement from preoperative to postoperative scores after repair, with no differences between repairing methods. We recommend simplifying the classification of ramp lesions as stable or unstable to determine treatment. Biological treatment is preferred for stable lesions rather than leaving them in situ. Unstable lesions, on the other hand, require repair, which has been associated with excellent functional outcomes and healing rates.

## Introduction and background

Ramp lesions of the medial meniscus occur when the meniscocapsular junction at the level of the posterior horn is injured, as first described by Strobel M in 1988 [1]. This region plays a crucial role in stabilizing the knee during anterior tibial translation, especially in the absence of the anterior cruciate ligament (ACL) [2]. Therefore, it is imperative to accurately diagnose and appropriately treat these injuries to restore knee stability. The incidence of medial meniscus injury in ACL lesions has been reported to range between 15% and 40% [3], with ramp lesions occurring in 9.3%-16.6% [4,5]. MRI can identify ramp lesions, but its sensitivity is not consistently high, with reported values ranging from 48% to 84.6% in different series [6,7]. Therefore, a thorough arthroscopic examination during surgery is essential to ensure proper diagnosis and treatment, particularly for these lesions. Two methods for intraoperative diagnosis have been described: first, a direct view of the posteromedial region through the notch [7], and second, an accessory posteromedial portal. Failure to perform these views during arthroscopy could result in the underdiagnosis of up to 40% of these lesions [8]. Various management options have been proposed for ramp lesions, including surgical and non-surgical treatments [9-12], with lesion stability being an important factor in selecting the appropriate technique. Stable lesions, defined as those smaller than 1 cm, do not require specific treatment [13-15]. Unstable lesions, defined as those larger than 1 cm or exhibiting anterior pathological translation of the posterior horn, necessitate repair using an accessory posteromedial portal or classic anterior portals. The all-inside suture technique was first described by Ahn JH et al. in 2004 for repairing the posterior horn of the medial meniscus via an accessory posteromedial portal, with a reported 97% healing rate in second-look arthroscopies after an average 19-month follow-up [9]. Currently, there is no consensus on the optimal management of ramp lesions of the medial meniscus, and there is a lack of an algorithm to determine the best approach for stable or unstable lesions. This systematic review aims to compare the clinical results of different surgical treatments for ramp lesions, including leaving the lesion in situ, meniscal repair, and biological treatments. The latter include abrasion or trephination. These biological treatments focus on harnessing the body's natural healing mechanisms by stimulating tissue regeneration and encouraging a healing response. We hypothesize that stable lesions have a favorable prognosis with techniques that do not require the use of meniscal sutures. In contrast, unstable lesions require appropriate fixation, either through an anterior or posteromedial portal.

## Review

Methods

**Figure 1:**
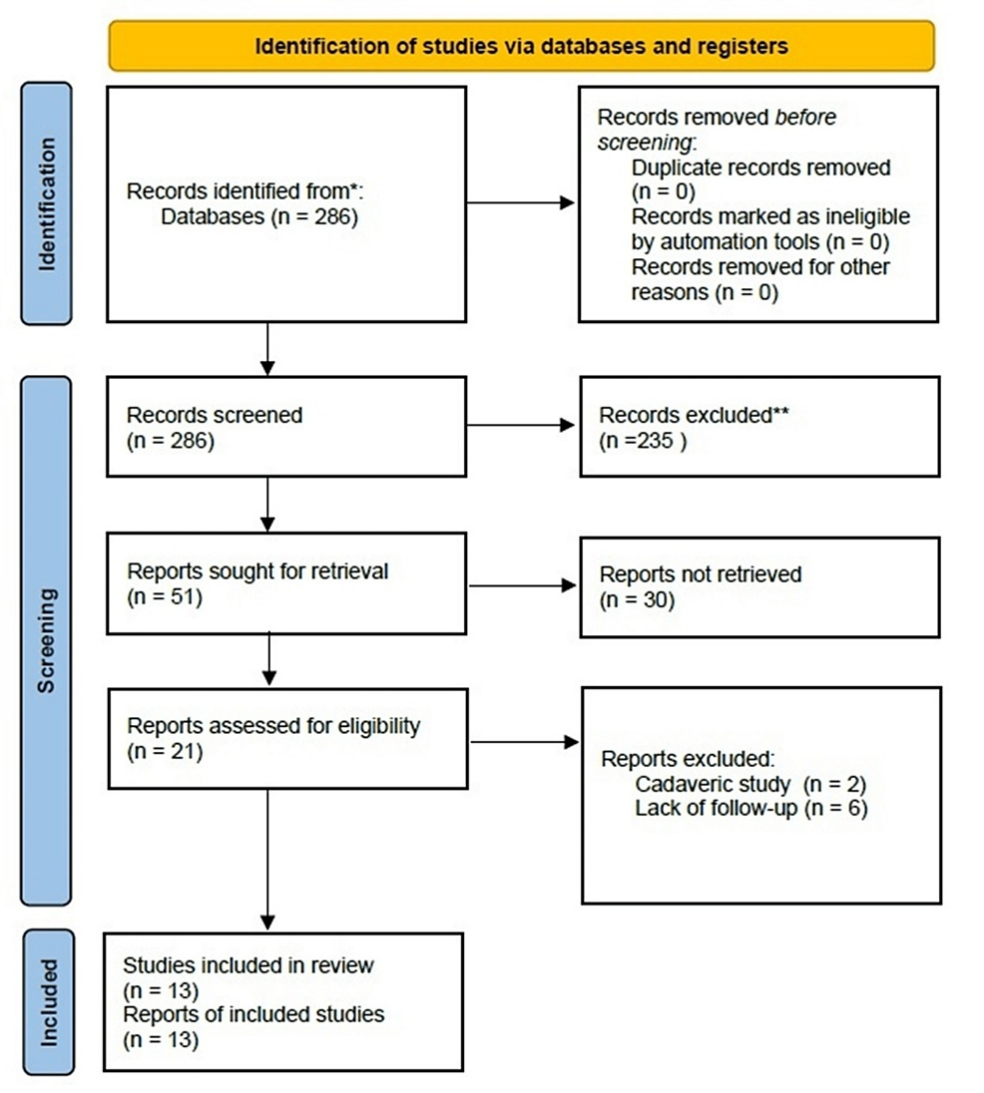
Flow chart outlining the study selection process as per PRISMA guidelines. PRISMA: Preferred Reporting Items for Systematic Reviews and Meta-Analyses. Source: [16]

A systematic literature search was designed. The study used Preferred Reporting Items for Systematic Reviews and Meta-Analyses (PRISMA) guidelines for a systematic review of clinical studies reporting outcomes of ramp lesion treatment [16]. In the MEDLINE/PubMed database, a search was performed using the following criteria: Mesh: ((ramp lesion) AND (medial meniscus ramp lesion)) OR (meniscocapsular injury) and non-Mesh: ramp lesion, ramp, meniscocapsular. Clinical studies on the treatment of ramp meniscal lesions, with a follow-up of at least six months with a report of functional results, clinical stability tests, radiological evaluation, or arthroscopic second look, published in English or Spanish, were included. Biomechanical studies, cadaveric studies, analysis of meniscal lesions other than ramp, technical notes without clinical results, and reviews were excluded. In the first search, studies were pre-selected based on the abstract by the author, downloading the full article for a final decision. The second and third authors were consulted to review the selected articles and obtain consensus in case of disagreement (Figure 1). All demographic data, surgical techniques, and treatment results of ramp lesions were extracted and included in the analysis.

**Figure 2:**
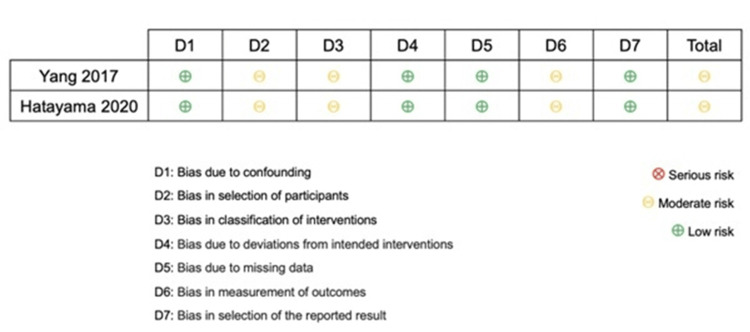
Summary of risk of bias in randomized trials. Source: [17]

**Figure 3:**
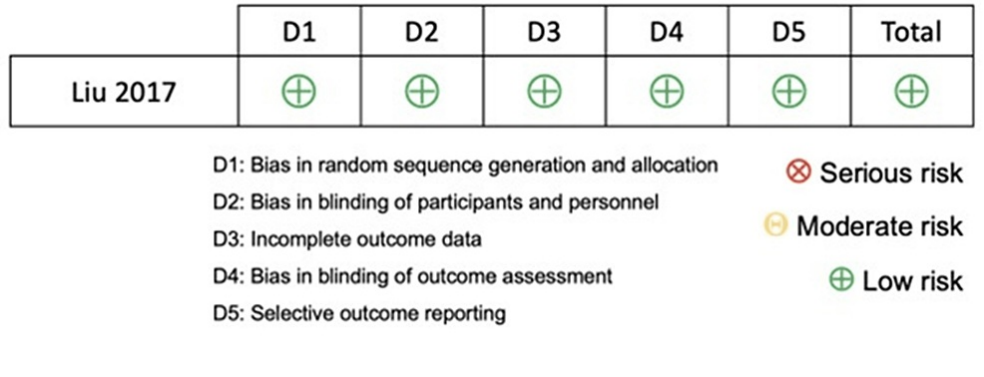
Summary of risk of bias of non-randomized trials. Source: [18]

A network meta-analysis was performed for the statistical analysis to compare three treatment alternatives for stable lesions: no treatment, biological treatment, and meniscal repair. Network meta-analysis is a statistical method that allows the comparison of multiple interventions in a network of studies, even if direct comparisons are lacking. Biological treatment refers to techniques that stimulate healing or regeneration, such as trephination, abrasion, or curettage. Two models were constructed with two outcomes: functional results assessed using the Lysholm score and failure to heal evaluated with MRI in follow-up. The reference "no treatment" was used in both models. The consistency of the models was assessed using the Wald test. If the probability of the test was greater than 0.15, the null hypothesis for consistency was accepted. Subsequently, the probability of being the best treatment and the surface under the cumulative ranking (SUCRA) were estimated for each treatment in each model. SUCRA is a measure that summarizes the rank of each treatment in terms of effectiveness, with higher scores indicating more effective treatments. Due to the lack of direct comparisons, a meta-analysis could not be performed to treat unstable ramp lesions. Instead, a critical analysis of the available literature was conducted. The bias of the studies included in the network meta-analysis was evaluated using the Cochrane tools for Rob-II randomized studies [17] and for non-randomized ROBINS-I studies (Figures 2-3) [18].

Results

The initial search yielded a total of 279 studies. After excluding duplicates and those unrelated to the required criteria, we identified 51 studies and analyzed their abstracts. Application of the inclusion criteria further narrowed down this selection, resulting in the inclusion of 13 studies. The analyzed population comprised 1614 patients, including 1122 men and 379 women. The sex of participants was not reported in one study with 23 patients [11]. The average age of patients was 30 years (range 18-75 years) in the nine studies that provided this information. The follow-up duration ranged from 6 to 47 months. The BMI of 132 patients from five studies [19-23] was documented, with an average of 24.7 (range 23.7-27.5). Only three studies [19,24,25] reported the time from injury to surgery, with the time range between injury and surgery being 8.3 to 97.1 weeks. The type of treatment varied between stable and unstable ramp lesions. Six studies differentiated between stable and unstable lesions based on criteria such as anterior displacement of the meniscus using a probe and lesion size [9,19,20,24-28]. For stable lesions, 90 cases received no treatment, 64 cases were treated biologically through techniques like abrasion, debridement, edge curettage, and trephination, and 728 cases underwent meniscal repair. Of those, 949 ramp injuries were repaired; 221 cases were unstable. Among the repaired cases, 340 patients underwent repair using an anteromedial portal with all-inside suture devices [9,11,20,23,26-30], while 609 patients were repaired through an accessory posteromedial portal in four studies (Table 1) [21,22,24,25].

**Table 1:** Summary of functional results, failure, and stability for each of the included studies. IKDC: International Knee Documentation Committee Subjective Knee Form.

Stable RAMP lesions													
Study	Treatment	n	Tegner	IKDC	SD	Lysholm	SD	MRI failure	Arthroscopic failure	High grade	Pivot Shift	High grade	Lachman test
Keyhani S et al. (2016) [21]	Repair with Suture-Lasso (Conmed-Livatec) through posteromedial portal	128		82.1	3.5	87.8	3.9						
	Repair with suture hook (Linvatec) through two posteromedial portals	40		83.6	3.7	88.7	4.8	1		0			0
Liu X et al. (2017) [5]	Biological treatment, with abrasion and trephination	33		82.2	4.5	90.4	5,8	2		0			0
	No treatment, left in-situ	25	6			98.5		10		0			
Hatayama K et al. (2020) [24]	Repair with Accu-Pass (Smith & Nephew) through posteromedial portal	21	6.8			98.7		0		1			
Albayrak K et al. (2020) [19]	No treatment, left in-situ	33		77.4	9.2	86	6.4			6			2
Jiang J et al. (2020) [29]	All-inside repair with OMNISPAN (DePuy-Synthes-Mitek) through anteromedial portal	20	85.7			85.7	3.5						
Balazs GC et al. (2020) [20]	No treatment, left in-situ	32		85.6	10.8								
Chen Z et al. (2017) [27]	All-inside repair with Fast-Fix (Smith & Nephew) through anteromedial portal	46		90.6	80-98	94.4	90-99		1				
Furumatsu T et al. (2014) [28]	All-inside repair with Fast-Fix (Smith & Nephew) through anteromedial portal	20				93.1	3.1		0				
Sonnery-Cottet B et al. (2018) [22]	Repair with Suture-Lasso (Arthrex) through posteromedial portal	416											
	All-inside repair with Fast-Fix (Smith & Nephew) through anteromedial portal	37		85.9	4	90.5	5.8	4					
Yang J et al. (2017) [26]	Biological treatment, curetage of the lesion margins ("refreshing")	31		85.1	4.2	90.3	8.7	4					

Unsable RAMP lesions													
Study	Treatment	n	Tegner	IKDC	SD	Lysholm	SD	MRI failure	Arthroscopic failure	High grade	Pivot shift	High grade	Lachman test
Li WP et al. (2017) [11]	All-inside repair with Fast-Fix (Smith & Nephew) through anteromedial portal	23				91.2	4.6						
Hatayama K et al. (2020) [24]	Repair with Accu-Pass (Smith & Nephew) through posteromedial portal	4	6.8			98.7		0			1		
Balazs GC et al. (2020) [20]	All-inside repair with Fast-Fix (Smith & Nephew) through anteromedial portal	23		80.6	17.2								
Ahn JH et al. (2004) [9]	Repair with suture hook (Linvatec) through two posteromedial portals	39				95.6	4.52		1				
Thaunat M et al. (2016) [23]	Repair with Suture-Lasso (Arthrex) through posteromedial portal	132	6.9	85.7	12								

Stable Ramp Lesions

**Figure 4:**
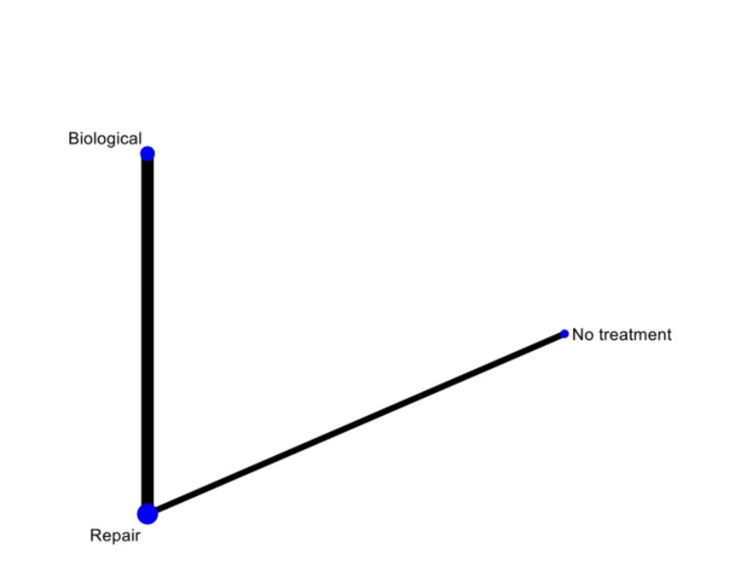
Network plot based on the Lysholm scale for stable meniscal ramp lesions.

The network meta-analysis included three studies [24-26] with 187 cases: 25 cases of no treatment, 64 cases of biological treatment, and 98 cases of meniscal repair. Hatayama K et al. [24] and Yang J et al. [26] had a moderate risk of bias in patient selection, intervention classification, and outcome measurement. In contrast, Liu X et al. [25] had a low risk of bias in all evaluated areas. Functional results of different treatments (no treatment, biological treatment, and meniscus repair) were compared using a network diagram. Only one indirect comparison was made between biological treatment and no treatment. Direct comparisons were made between no treatment versus meniscal repair (based on one study) and biological treatment versus meniscal repair (based on two studies). The network diagram helped visualize the relationship between different treatments and their direct and indirect comparisons (Figure 4).

**Figure 5:**
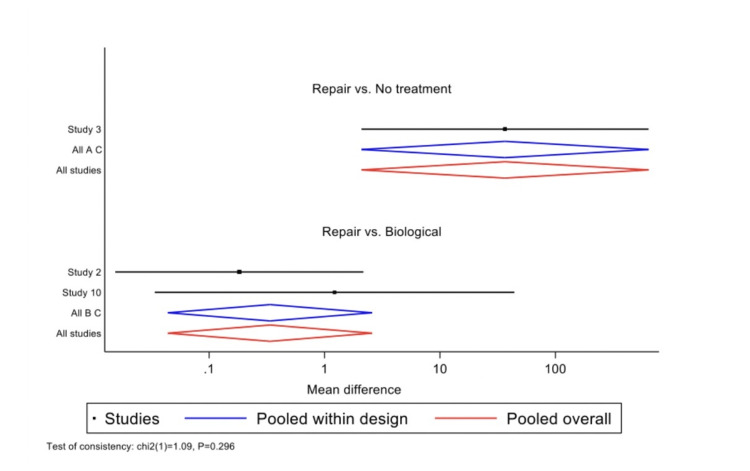
Effect size plot based on Lysholm scores.

**Figure 6:**
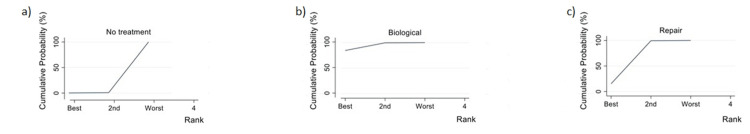
SUCRA of each treatment using Lysholm as outcome. SUCRA of no treatment (a), biological treatment (b) and repair treatment (c) using Lysholm as outcome. SUCRA: Surface under the cumulative ranking.

**Figure 7:**
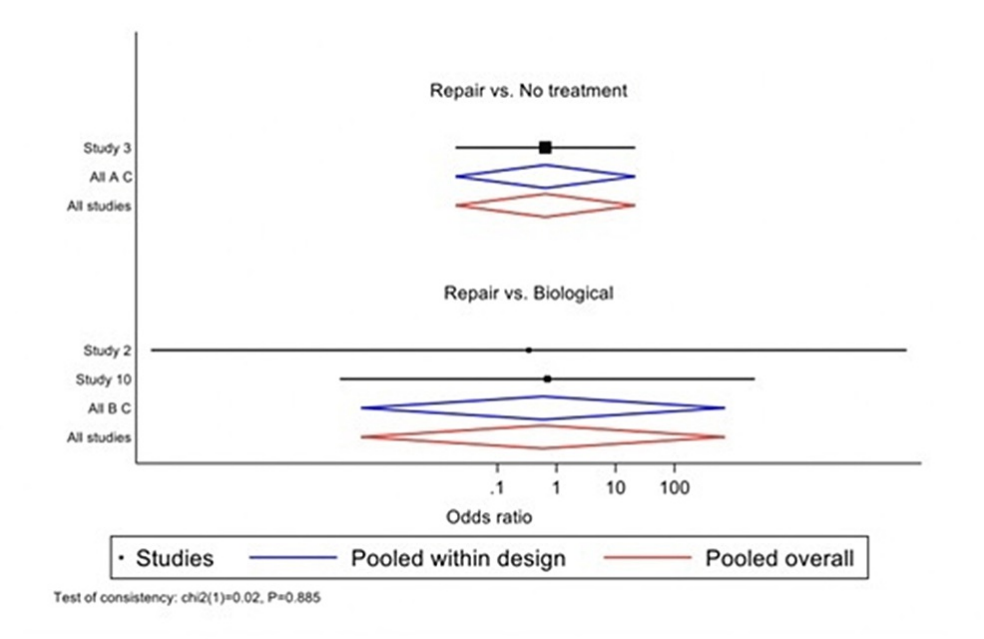
Effect size plot indicating failure as determined by MRI.

**Figure 8:**
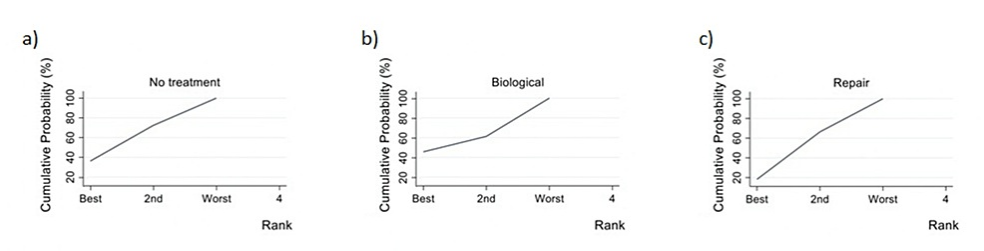
SUCRA of each treatment using failure on MRI as outcome. SUCRA of no treatment (a), biological treatment (b) and repair treatment (c) using failure on MRI as outcome. SUCRA: Surface under the cumulative ranking.

Wald's inconsistency test accepted the null consistency hypothesis in both models. In the Lysholm model, "repair" had a coefficient of 3.60 (p=0.014), and "biological treatment" had a coefficient of 4.69 (p=0.009). In the MRI model, "repair" had a coefficient of -0.433 (p=0.809), and "biological treatment" had a coefficient of 0.09 (p=0.982). When assessing the outcome using the Lysholm score, the best-estimated treatment was "biological," with a SUCRA of 0.9 and a probability of being the best treatment of 0.846. "Repair" ranked second with a SUCRA of 0.6, and "no treatment" ranked third with a SUCRA of 0 (Figures 5-6, Table 2). When assessing the outcome using MRI failure, the best-estimated treatment was again "biological," but with a lower SUCRA of 0.5 and a probability of being the best treatment of 0.45. In this case, "no treatment" ranked second with a SUCRA of 0.5, and "repair" ranked third with a SUCRA of 0.4 (Figures 7-8, Table 3).

**Table 2:** Probabilities of each treatment ranking as best, second best, or worst, based on the Lysholm scale as the primary outcome. SUCRA: Surface under the cumulative ranking.

Lysholm	Best	Second	Worst	Mean rank	SUCRA
No treatment	0.2	0.7	99.2	3	0
Biological	84.2	15.1	0.3	1.2	0.9
Repair	15.2	84.2	0.5	1.9	0.6

**Table 3:** Probabilities of each treatment ranking as best, second best, or worst, based on failure on MRI as the principal outcome. SUCRA: Surface under the cumulative ranking.

Failure on MRI	Best	Second	Worst	Mean Rank	SUCRA
No treatment	36.2	36.2	27.6	1.9	0.5
Biological	45.6	15.7	38.7	1.9	0.5
Repair	18.2	48.1	33.6	2.2	0.5

Unstable Ramp Lesions

Three functional scores were used to evaluate the results of unstable injuries: the International Knee Documentation Committee (IKDC) [19-21,23,25-28], Lysholm [9,11,19,21,24-30], and Tegner [23,25,30]. All seven studies that assessed IKDC scores demonstrated a significant improvement from preoperative to postoperative scores. However, only one study made a direct comparison between the outcomes of different treatments. Balazs GC et al. [20] evaluated stable lesions left in situ and unstable lesions repaired by an anteromedial portal with an all-inside suture, obtaining scores of 85.6 and 80.6, respectively. The difference was not statistically significant despite the higher IKDC value in the stable lesion group. Regarding the Lysholm score, all studies that reported on it showed a significant improvement between preoperative and postoperative scores. However, no significant differences were noted when comparing various treatment groups. For instance, Hatayama K et al. [24] compared unrepaired stable lesions with unstable lesions repaired by the posteromedial portal, but no significant differences were found. Tegner scores were reported by Thaunat M et al. [23]. They noted a slight decrease from 7.2 to 6.9 (p<0.0017) between pre-injury scores and post-surgery scores when the ramp lesion was repaired. In a comparison of postoperative Tegner scores between two treatment approaches, Hatayama K et al. [24] reported a score of 6.0 in the unrepaired group and 6.8 in the repaired group. MRI was used in two studies to assess healing, with 107 lesions (86.99%) showing complete healing, including 58 repaired, 29 treated with biological treatment, and 10 left in situ [24,25]. Thirteen lesions were reported as unhealed, including 10 stable injuries left in situ, one requiring repair, and two treated with biological treatment. Hatayama K et al. [24] performed an MRI evaluation at 12 months for repaired unstable lesions, with 30 lesions showing complete healing (20 repaired, ten left in situ) and ten lesions without healing (all left in situ). Three studies evaluated the quality of healing of the ramp injury with second-look arthroscopy [9,28,29]. The average time between the primary surgery and the second look ranged from 14 to 32 months. All studies used similar criteria to define complete or incomplete healing and failure. Complete scarring was defined as no palpable injury or a small indentation of less than 10% of the meniscal thickness. Failure was defined as a cleft of 50% or more, meniscal thickness compromise of more than 50%, or instability when probed. Ahn JH et al. [9] performed 39 second-look arthroscopies, demonstrating 82.1% complete healing, 15.4% incomplete healing, and 2.6% failure. They emphasized that all cases of complete scarring were in the posterior horn at the site where all-inside sutures were performed, while incomplete scarring occurred with inside-out sutures.

Considerations

The main finding of our study is that stable lesions that do not show displacement with a probe benefit from biological treatment, which appears to be superior to leaving them in situ. Furthermore, the best treatment for unstable injuries would be repair, regardless of the technique. The biological treatment described by Liu X et al. [25] and Yang J et al. [26] includes techniques such as abrasion, trephination, regularization of the edges, and "shrinking." These augmentation techniques are often used to enhance the outcomes of meniscal sutures by providing cells and fibrin clots that promote healing. Similar techniques have shown favorable results when used in meniscal repairs, especially when combined with ACL reconstruction [31]. While our study suggests that all ramp lesions should be treated with biological treatment for stable lesions and repair for unstable lesions, it is worth mentioning the results of Shelbourne KD and Heinrich J's study [31]. They propose not repairing meniscus lesions in the posterior horn and root of the lateral meniscus and achieved good results with only a 3.3% failure rate requiring subsequent meniscus surgery. Based on these principles and considering that ramp lesions are in the meniscocapsular junction, some authors suggest this approach for cases without mechanical instability. Liu X et al. [25], in their prospective study, obtained comparable results between abrasion/trephination and repair for stable ramp lesions in terms of the Lysholm score, IKDC, stability, and MRI healing evaluation. They highlight the importance of achieving anteroposterior stability after ACL surgery, as it can affect the outcomes [25]. Yang J et al. [26] also compared edge debridement versus repair as treatments and found that edge debridement resulted in shorter surgery duration, faster postoperative functional recovery, and shorter hospital stays. MRI evaluation showed no significant differences [26]. Our findings indicate that stable lesions left in situ have lower outcomes than biological treatments, prompting us to consider direct observation with the trans-notch view (i.e., the modified Gillquist maneuver) to detect possible ramp lesions [32]. Direct observation remains the primary diagnostic method, as MRI has a sensitivity range of 53.9%-84.6%, and specificity range of 92.3%-98.7%, with a negative predictive value range of 91.1%-97.4%, and a positive predictive value range of 50%-90% [6]. DePhillipo NN et al. [33] reported that 11% of surveyed surgeons do not use MRI as a preoperative diagnostic method, and 22% mentioned that MRI accuracy is often limited. More than half (56%) of the respondents considered posteromedial bone edema an important secondary finding suggesting a ramp lesion [34]. Follow-up MRI for these treated lesions may not be useful due to the absence of evaluation criteria and limited literature. In the analyzed series of our study, no differences were observed between repaired and unrepaired lesions. Treatment failure should be clinically analyzed, with persistent meniscal symptoms and medial joint line tenderness being the main indicators [21]. Regarding the criterion of instability, among the studies analyzed, probe manipulation and anterior translation of the meniscus appear more important than the lesion size. Generally, a 1 cm length criterion is used for meniscal sutures in various types of lesions, which is also extrapolated to ramp lesions. However, some authors propose not repairing lesions larger than 1 cm if they do not displace with the probe [34]. Shelbourne K and Rask BP [35] reported treating 94% of 325 asymptomatic patients with simple abrasion and trephination of the meniscal bed for stable lesions, regardless of size. In this systematic review, different criteria were used, but probe manipulation was commonly proposed as the method to determine stability rather than lesion length. Resolving this controversy is important because it determines whether meniscal repair should be performed. The existing Thaunat classification does not help define the treatment, as it is purely descriptive [20]. Based on our review, we believe it is more practical to classify ramp lesions as stable or unstable. DePhillipo NN et al. [33] evaluated the practices of 36 knee surgeons and sports medicine specialists through a questionnaire. They found that 89% considered the extent of the lesion (partial or complete) as the primary stability criterion, while 81% used probe manipulation [34]. The heterogeneity in the definition of stable or unstable lesions is a significant limitation of this review, emphasizing the need for further discussion and consensus on defining and classifying meniscal injuries. The lack of a standardized definition of stability or instability can make it challenging to apply the findings of this review to clinical practice. Another limitation of our study is that we only searched for articles in Spanish and English and only in one database, which may have excluded relevant studies from other sources or in other languages. This limitation could have introduced bias in the selection of studies included in the meta-analysis and may restrict the generalizability of our findings. Additionally, there is a limitation due to the lack of comparative studies in stable lesions, which prevented a meta-analysis within this subgroup. Moreover, the quality of the included studies was generally low, with most of them presenting type IV evidence. Furthermore, two studies with a moderate risk of bias were included in the meta-analysis, which decreased the power and quality of the analysis. In addition, there is heterogeneity among the included studies regarding patient characteristics, lesion location, size, and stability criteria. This heterogeneity may have influenced the overall results and limited the generalizability of our findings. Lastly, in postoperative functional scores (e.g., Lysholm), isolating the effect of anterior cruciate ligament reconstruction is impossible. Therefore, further studies are needed to confirm our findings and determine the optimal treatment for ramp lesions based on patient and lesion characteristics.

## Conclusions

In conclusion, our study highlights the importance of a simplified classification of ramp lesions of the meniscus into stable and unstable categories, as it provides guidance for appropriate treatment decisions. For unstable injuries, repair with sutures following manipulation with the probe is recommended. On the other hand, stable lesions can benefit from biological treatments such as abrasion, trephination, and curettage of edges, which promote and facilitate the healing process of the lesion. However, it is essential to acknowledge the limitations of our study. The heterogeneity in the definition of stability or instability among the analyzed studies underscores the need for further consensus and discussion on defining and classifying meniscal injuries. Additionally, the low quality of the included studies, the limited number of comparative studies in stable lesions, and the language and database restrictions in our search may have introduced bias and limited the generalizability of our findings. Further research is warranted to validate our findings and determine the optimal treatment approach for ramp lesions based on patient and lesion characteristics. Additionally, standardized criteria for stability assessment and comprehensive evaluation tools are needed to improve clinical decision-making in managing these challenging meniscal injuries. In summary, our study provides valuable insights into treating ramp lesions of the meniscus, emphasizing the importance of distinguishing between stable and unstable lesions. This knowledge can aid clinicians in making informed decisions regarding repair or biological treatment, ultimately improving outcomes for patients with these specific meniscal injuries.
